# Destroying the Nerve in Decayed Teeth

**Published:** 1892-01

**Authors:** 


### DESTROYING THE NERVE IN DECAYED TEETH.

After cleansing and drying the cavity with pellets of cotton-wool wound round the end of a crochet needle, or the eye end of a darning needle, or any thing which has some kind of notch at the end to hold the cotton fast, then take another pellet of wool, about large enough to half fill the cavity, saturate it with carbolic acid, place it in the tooth, and cork it in with a small piece of the white or pink gutta-percha, softened in warm water, which is sold by druggists for the purpose of plugging decayed teeth. In a few hours all the nerve matter that the acid can get at will be destroyed. In some persons the gnawing pain caused by the process is very considerable, and it can be mitigated by applying a mixture of tincture of aconite and chloroform, procurable from a chemist of the right strength and proportions, to the tooth and gums adjacent. This treatment will destroy the nerves, and in most cases quite painlessly. But it will not arrest the caries, and in less than a week the heat and distress in the tooth will compel removal of the stopping to relieve tension. The best way by far, as the brittle nature of the teeth in this case precludes ordinary stopping, is to ask a dentist to cut away the carious dentine and insert a gutta-percha plug of the kind described, which, in favorable cavities will last for a year ; and when the gutta-percha gets worn or dislodged by mastication, it is an easy matter to have it renewed, or even renew it oneself, after the caries has been once removed by a dentist, and the patient has had the experience of one professional stopping.—R. A. C.

